# Giants, Dwarfs and the Environment – Metamorphic Trait Plasticity in the Common Frog

**DOI:** 10.1371/journal.pone.0089982

**Published:** 2014-03-05

**Authors:** Franziska Grözinger, Jürgen Thein, Heike Feldhaar, Mark-Oliver Rödel

**Affiliations:** 1 Department of Animal Ecology and Tropical Biology (Zoology III), Theodor-Boveri-Institute (Biocenter of the University), Würzburg, Germany; 2 Museum für Naturkunde Berlin, Leibniz Institute for Research on Evolution and Biodiversity, Berlin, Germany; 3 Büro für Faunistik und Umweltbildung, Hassfurt, Germany; 4 Animal Ecology I, Bayreuth Center of Ecology and Environmental Research (BayCEER), University of Bayreuth, Bayreuth, Germany; 5 Berlin-Brandenburg Institute of Advanced Biodiversity Research (BBIB), Berlin, Germany; Fred Hutchinson Cancer Research Center, United States of America

## Abstract

In order to understand adaptation processes and population dynamics, it is central to know how environmental parameters influence performance of organisms within populations, including their phenotypes. The impact of single or few particular parameters in concert was often assessed in laboratory and mesocosm experiments. However, under natural conditions, with many biotic and abiotic factors potentially interacting, outcomes on phenotypic changes may be different. To study the potential environmental impact on realized phenotypic plasticity within a natural population, we assessed metamorphic traits (developmental time, size and body mass) in an amphibian species, the European common frog *Rana temporaria*, since a) larval amphibians are known to exhibit high levels of phenotypic plasticity of these traits in response to habitat parameters and, b) the traits' features may strongly influence individuals' future performance and fitness. In 2007 we studied these metamorphic traits in 18 ponds spread over an area of 28 km^2^. A subset of six ponds was reinvestigated in 2009 and 2010. This study revealed locally high variances in metamorphic traits in this presumed generalist species. We detected profound differences between metamorphing froglets (up to factor ten); both between and within ponds, on a very small geographic scale. Parameters such as predation and competition as well as many other pond characteristics, generally expected to have high impact on development, could not be related to the trait differences. We observed high divergence of patterns of mass at metamorphosis between ponds, but no detectable pattern when metamorphic traits were compared between ponds and years. Our results indicate that environment alone, i.e. as experienced by tadpoles sharing the same breeding pond, can only partly explain the variability of metamorphic traits observed. This emphasizes the importance to assess variability of reaction norms on the individual level to explain within-population variability.

## Introduction

Organisms often respond to changing environments by altering life-history traits and even morphology [1], [2]. When a single genotype produces different phenotypes in dependence of the environment, this is termed phenotypic plasticity [3]. This plasticity is adaptive if the expression of alternative phenotypes allows an organism to exploit a wider range of environments [4], [5]. In order to understand the long-term consequences of habitat alteration, i.e. due to climatic shifts or anthropogenic actions, it is crucial to study the response of organisms to environmental parameters [6].

Experimental setups in laboratories and mesocosms are often used to infer effects of different factors on phenotypic plasticity. While the advantage of such experiments is the reduction of environmental noise, recent findings show that not only the magnitude but also direction of organisms' responses can differ between experimental venues [7], [8]. Therefore, studies of natural populations are needed to validate the influence of particular parameters.

Larval amphibians are perfectly suited to test the influence of environmental parameters on their development since they respond to different environmental conditions with a high plasticity in morphology, behaviour and development, and consequently metamorphic traits [9], [10]. Size and mass at metamorphosis considerably influence juveniles' mobility, predation risk, and survival [10]–[12], as well as age and size at maturity and thus fecundity [13]–[15]. Therefore, metamorphic traits are of crucial importance not only for an individual's future performance and fitness, but also for the dynamic and survival of local populations [16], [17].

The influence of particular environmental parameters on larval development time and metamorphic mass are well studied under laboratory conditions. There, focus was laid on the influence of biotic and abiotic factors such as population density [18], [19], predators [20], [21], pH [22], [23], desiccation risk [24], [25] or food availability [26], [27]. However, even on distances of only a few hundred meters [28], experimental data on phenotypic plasticity in natural populations reveal considerable divergence in behaviour, morphological and metamorphic traits [29], [30]. Additionally, alteration of a single factor such as absence or presence of predators in experimental setups yields contradictory metamorphic responses in different studies [21]. Under natural conditions, with many biotic and abiotic factors potentially interacting, outcomes on phenotypic changes may be even less predictable.

In order to investigate the influence of a variety of abiotic and biotic factors on phenotypic plasticity, we studied the metamorphic traits (development time, size and mass at metamorphosis) of the European common frog, *Rana temporaria*, under natural conditions. *Rana temporaria* is an excellent species to study environmental influence on individual performance since it shows high plasticity in morphology, behaviour and larval developmental traits (e.g.[28]).

Our study population is known to exhibit strong breeding site preferences for particular ponds, and avoiding many others [31]. Since developmental habitat has a strong impact on fitness of amphibians we asked: a) if chosen ponds actually are suitable habitats, i.e. do larvae survive, b) if there are differences in the performance of tadpoles (development time, metamorphic mass), between ponds with metamorphing frogs, and c) if these differences can be related to environmental features of the ponds.

## Materials and Methods

### Ethics Statement

No Institutional Animal Care and Use Committee (IACUC) or ethics committee approved this study as this was not required by German law. According to the German Protection of Animal Act (“Tierschutzgesetz”, latest adapted on 9 December 2010; http://www.gesetze-im-internet.de/tierschg/BJNR012770972.html; assessed on 12 June 2013) painless experiments and observations with vertebrates neither require permission nor disclosure (§ 1/§ 7 TierSchG). The vertebrates involved, *Rana temporaria*, experienced no pain, suffering, complaints or harm. Peter Krämer (Regierung von Unterfranken) approved the research in accordance with the “Conservation of Nature and of Landscape Act” (Federal Nature Conservation Act). Ulrich Mergner (Bayerische Staatsforsten) permitted work in the forests under his care. All our work complied with the guidelines for the use of live amphibians and reptiles in field research compiled by the American Society of Ichthyologists and Herpetologists (ASIH), The Herpetologists' League (HL) and the Society for the Study of Amphibians and Reptiles (SSAR).

### Study site and species

We conducted our study within a 28 km^2^ forested area in northern Bavaria, Germany (49°55′N, 10°33′E). There, we continuously monitored more than 70 ponds for breeding site use by *Rana temporaria* since 2005 [31]. Between March and October 2007 we investigated developmental success of *R. temporaria* in 18 ponds, beginning with spawning and ending with the last emigrating juveniles. Selected ponds varied in breeding activity (clutch numbers), size and other characteristics (see [31], Table S1).

*Rana temporaria* is a widespread Eurasian frog, ranging between northern Spain, northern Scandinavia and beyond the Ural into Siberia [32]. Within a region, spawning usually takes place within a few days in early spring. A multitude of different habitats are used for breeding [32]. In our study area the species breeds in forest ponds which are of small to medium size and semi-temporary, drying out irregularly [31].

### Characterisation of pond parameters

Pond characteristics were determined on 23 and 24 May 2007, if not specified otherwise (Table S1), when vegetation was completely developed. These characteristics comprised all parameters, known from other studies to be of potential influence on tadpole development: i.e. physical (pond volume, water depth, temperature) and chemical (pH, nitrate, phosphate), as well as various vegetation parameters (e.g. canopy openness, duckweed cover) of the ponds ([31] for details). A total of 18 pond characteristics (Table S1) were grouped as ‘abiotic factors’ while density of predators and tadpoles were referred to as ‘biotic factors’. Apart from *R. temporaria* tadpoles, no other anuran larvae were observed in the ponds.

To estimate predation pressure and intraspecific competition during larval development, density of *R. temporaria* tadpoles and their predators was measured three times between 24 April 2007 and 1 June 2007 using box sampling [33]. Per sampling period we collected one sample (box length × width × height: 50×50×80 cm) for appr. every 10 m^2^ of pond surface. In order to avoid random effects in small ponds and to reasonably limit handling time in large ponds, we tried to get a minimum of two box samples in small ponds (possible for all but one sampling event at one pond), and a maximum of eight samples for large ponds. Samples were equally distributed with regard to shallow (up to 0.5 m distance from shore) and deeper water, keeping a minimum distance of 2 m between two sample sites (see [33]). All tadpoles and all potential tadpole predators trapped in a box were counted and released. Based on the water volume in the box, the number of specimens per taxon, and the ponds' volumes, we calculated the mean density of each taxon per pond and sampling date. We grouped predator- and tadpole-density over the three sampling events in further analyses.

We defined dragonfly larvae, adult newts (*Ichthyosaura alpestris, Lissotriton vulgaris*), water frogs (*Pelophylax* spp.), Notonectidae and Dytiscidae (imagines and larvae) with a minimum size of 1.5 cm as potential predators, since they are known to prey on *R. temporaria* larvae [28], [32]. Non-independent effects of multiple predator species were reported in several predator-prey systems [34], [35]. For predators present in our study system, however, Ramos and Van Buskirk [36] reported no interaction effects between backswimmers, newts and dragonfly larvae, and a constant mortality rate per predator for backswimmers (*Notonecta* sp.) and newts (*I. alpestris*), independent of predator-density. In an experimental setup dragonfly larvae proved to be more effective in killing *R. temporaria* tadpoles than other predators [28], but also to interfere with each other, thus lowering their predation rate when occurring in higher densities [36]. Therefore, we considered the use of overall predator-density being the most appropriate surrogate for predation risk in our study, neglecting the potential differences in relative dangerousness of predator species [28], [37].

During the study period, some ponds showed intensive water loss. One pond lost all open water, however, some tadpoles survived in deep and moist leaf litter until rain filled the pond again (AW04). Low water levels affected temperature measurements as well as the assessment of predator- and tadpole-density. Thus, these parameters could not be measured for all ponds at all events. Missing temperature values were substituted by overall temperature mean of the remaining ponds to keep this variable in the analysis. Analyses including biotic parameters could only be measured for emigration ponds (except AW04).

### Tadpole development and survival

To assess the initial number of eggs deposited in a particular pond, ponds were screened three times during the breeding season (20 and 27 March 2007, 3 April 2007) and clutches were counted individually. As newly deposited clutches can be easily differentiated from clutches being already present in the pond for a day or more, double counting of clutches was no problem. If several clutches were fused to an indistinguishable mass, we estimated the clutch number by using the surface of a hand as a rough guide for one clutch [31]. Survival rate was calculated as ratio of emigrating juveniles to eggs in the pond. Since egg numbers per clutch can vary profoundly (600–4000 eggs [32]), we determined egg numbers of 13 clutches of the study population (mean ± SD: 1117±321 eggs/clutch) and used the average for survival calculation. We regularly monitored all study ponds for tadpoles. As soon as front limbs began to break through, ponds were entirely fenced (8 and 12 June 2007) leaving sufficient space (>15 cm) between water line and fence for the metamorphs to emigrate from water. Starting at 13 June 2007, each pond where metamorphs emigrated was visited five to six times a week. Juveniles were collected at the pond side of the fences and transported to the laboratory for measurements. All measured juveniles were about finishing metamorphosis (Gosner stages 45–46), indicated by very small tail stubs (necrotic tissue, <1 mm). We determined the development time (time from oviposition to metamorphosis), the total number of emigrating juveniles per pond and visit, snout-vent-length (SVL; ±0.05 mm), and mass (± 0.002 g). Time of metamorphosis was consistent with time of emigration from ponds and thus data collection. If more than 50 juveniles were encountered at a particular pond and visit, size and mass of a randomly chosen subsample of 30 juveniles were taken. Due to higher precision in measurement, we used mass as a surrogate for metamorphic size in further analyses. After measurements, juveniles were released at the forest side of the fence at their pond of origin.

Using the same procedure as in 2007, five of the study ponds were surveyed again for metamorphic traits in 2009 and 2010; i.e. recording number of clutches, as well as number, development time, size, and metamorphic mass of emigrating juveniles.

### Statistical analysis

In natural systems, a variety of different parameters interact, hampering the detection of patterns. Therefore, we used different statistical approaches to explore potential patterns, concerning different questions with regard to environmental influence on tadpoles' developmental traits. To exclude a spatial autocorrelation of environmental parameters [38], we first applied a Mantel-test [39] which verified the independence of environmental similarity (habitat parameter values, see above) and geographic distance between ponds (Mantel-test, r = 0.057, p = 0.251, 999 permutations, based on Euclidean distance).

Second, we elucidated environmental patterns explaining survival in the ponds. We applied a principal component analysis (PCA) to environmental parameters to summarize the data. We used the axes explaining most of the variance of the data in a logistic regression to analyse the survival ratio in the ponds. Furthermore, we applied different grouping-techniques to discriminate between emigration and non-emigration ponds: we performed a hierarchical clustering with all 18 study ponds and the respective scaled values for their abiotic habitat parameters. The clustering was based on Euclidean distance, using Ward's minimum variance clustering for defining groups in order to minimize the within-group sum of squares [40]. We further used bootstraps (1000 runs) to estimate the accuracy of the cluster. Thereafter, we applied k-means clustering, which allows a predefinition of number of groups. There, we used the Hartigan and Wong [41] algorithm with 20 randomized starts to group the ponds into two clusters, based on scaled abiotic parameters. Additionally, we used the machine learning algorithm ‘random forest’ [42], which proved to be a powerful statistical classifier with a high prediction accuracy, in order to determine variable importance and to model complex interactions among predictor variables [43], [44]. Random forest grows many binary classification trees, merges the derived predictions and evaluates the importance of variables for the decision (for a detailed description of the method see [42], [43]).

Third, we related metamorphic traits to pond characteristics. Thus, PCA-axes of pond characteristics were used as explanatory variables in linear regressions to explain metamorphic mass, size and development time. During our analysis, we observed considerably different ‘emigration patterns’ i.e. the plot of metamorphic mass of each emigrating juvenile over its emigration date. To elucidate the basis for this divergence in derived curve shapes, we carried out an additional analysis using quadratic regression to describe the relationship between metamorphic mass and development time in the ponds.

We used the confidence intervals of the regression coefficients to group ponds and compare their habitat characteristics in order to look for similarities in emigration pattern and habitat. Subsequently, in order to identify the environmental parameters which best describe the observed pattern of metamorphic mass over time in the ponds, we used model selection based on corrected Akaike information criterion (AICc [45], [46]). There, different models are simultaneously evaluated and ranked in respect of their support of the given data [47]. Statistical analyses were conducted using the packages ‘nlme’ [48], ‘MASS’[49], ‘vegan’[50], and ‘MuMIn’[51] in R version 2.15.2 [52].

## Results

### Pond characterisation

**Figure 1:**
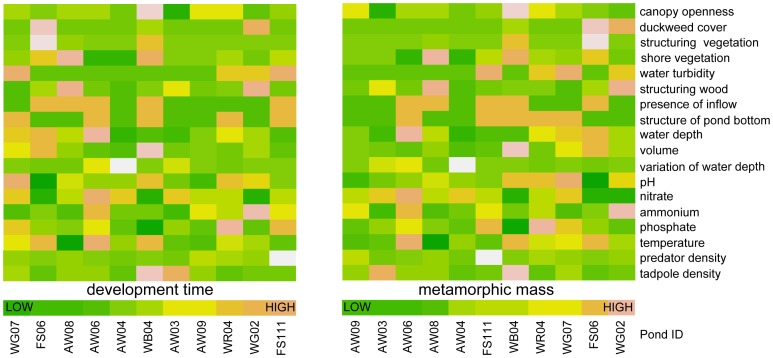
Pond characteristics and metamorphic traits. Visualization of environmental parameters in relation to *Rana temporaria* metamorphic mass (median) and development time (median). Values are colour-coded, warm colours indicating higher values. Ponds (bottom) are ordered according to their values for the respective trait, the values for the respective environmental parameter are given in the column above. There is no correlation between traits and environmental parameter visible (rows).

Ponds varied considerably in their abiotic characteristics, e.g. canopy openness ranged from 10.6 to 33.0% and water depth was between 7 and 57 cm. Two parameters (saprobel, submerged vegetation) were excluded due to lacking variance in parameter values, resulting in 16 abiotic parameters in further analysis (Figure 1, Table S1). There was no significant correlation of environmental parameters and mean metamorphic traits, except for duckweed cover and nitrate detectable (see Table S2). In general, predator- and tadpole-density were highly variable, both between visits and ponds (Table 1). Predator-density (predators per m^3^: p/m^3^) in ponds differed significantly between visits (sampling 1: 244.44 p/m^3^±376.45, n = 13; sampling 2: 33.24 p/m^3^±29.30, n = 17; sampling 3: 34.22 p/m^3^±72.48, n = 18; Friedman-Test, χ^2^ = 14.39, df = 2, p<0.001) and was lower at the second sampling ∼110 p/m^3^ compared to the first (Wilcoxon-signed-rank-test, V = 84, p = 0.005). However, there was no difference in predator-density between second and third sampling (V = 35, p>0.05, see Table 1).

**Table 1:** Summary of metamorphing *Rana temporaria* juveniles and predator- and tadpole-density in the respective ponds in 2007.

pond	cl.			size [mm]				mass [g]				devel.time [d]				predators/m^3^	tadpoles/m^3^	
		metamorphs (total/meas.)	survival rate [%]	mean	min	max	CV	mean	min	max	CV	mean	min	max	CV	mean ± SD	mean ± SD	samp.
AC01	1	0/0	0.00	–	–	–	–	–	–	–	–	–	–	–	–	35.6±50.3	0.0±0.0	2/4
AW03	47	1157/888	1.69	11.3	9.4	14.4	0.06	0.14	0.08	0.24	0.15	112.5	84	174	0.13	11.1±19.2	5233.0±6997.9	3/6
AW04	4	32/32	0.72	13.6	11.4	17.6	0.12	0.24	0.14	0.5	0.39	106.3	79	152	0.20	0.0±0.0	11.1±15.7	2/3
AW06	67	275/269	0.36	11.5	9.6	16.7	0.09	0.15	0.09	0.44	0.29	101.8	82	141	0.14	35.0±4.5	1468.3±2366.2	3/6
AW08	53	1159/868	1.47	11.9	9.5	17.6	0.11	0.17	0.08	0.49	0.38	104	82	161	0.19	48.3±29.4	1487.1±762.8	3/6
AW09	39	1751/1368	3.14	11.4	9	15.2	0.09	0.14	0.07	0.32	0.28	115.9	82	196	0.23	95.8±87.4	1313.0±1761.8	3/6
FS06	22	857/696	2.83	14.7	11.5	17.9	0.07	0.32	0.12	0.55	0.20	100.2	82	159	0.16	12.8±15.2	126.3±154.3	3/7
FS111	20	83/79	0.35	13.4	11.3	16.8	0.08	0.24	0.15	0.44	0.21	124.2	94	172	0.12	540.4±670.2	418.1±251.7	3/6
RS04	3	0/0	0.00	–	–	–	–	–	–	–	–	–	–	–	–	202.6±311.5	0.0±0.0	3/9
RS04Rinne	5	0/0	0.00	–	–	–	–	–	–	–	–	–	–	–	–	0.0±0.0	0.0±0.0	1/2
RS06	7	0/0	0.00	–	–	–	–	–	–	–	–	–	–	–	–	51.0±35.9	0.0±0.0	3/6
RS08	21	0/0	0.00	–	–	–	–	–	–	–	–	–	–	–	–	33.3±47.1	0.0±0.0	2/4
RS09	2	0/0	0.00	–	–	–	–	–	–	–	–	–	–	–	–	248.0±345.5	0.0±0.0	3/10
WB04	190	593/492	0.23	13.5	11	16.7	0.08	0.25	0.13	0.45	0.25	111.5	83	163	0.17	13.9±8.2	7650.3±12824.2	3/25
WB07	105	0/0	0.00	–	–	–	–	–	–	–	–	–	–	–	–	0.0±0.0	0.0±0.0	2/4
WG02	20	128/124	0.56	15.6	12.6	20.5	0.08	0.39	0.2	0.7	0.23	122.9	90	169	0.11	41.7±33.5	635.7±1030.8	3/7
WG07	14	1206/820	5.24	13.9	11.2	21.4	0.07	0.27	0.11	0.7	0.18	95.7	79	170	0.17	42.1±18.2	1725.1±2426.0	3/9
WR04	12	107/99	0.74	13.3	10.8	18.5	0.09	0.26	0.14	0.6	0.25	120	90	169	0.14	64.3±27.7	72.5±77.6	3/7

Survival rate was based on an average of 1117 eggs per clutch. For metamorphic traits, mean, coefficient of variance (CV), as well as minimum and maximum size and mass of individuals for each pond is given. For predator- and tadpole-density, mean and SD of all samplings, number of sampling events and total number of box samplings summed up for realized samplings are given (see methods for detailed description). For pond abbreviations, see [31].

cl.  = clutches; meas.  = measured, devel.time = development time, samp.  = samplings: events/total samplings.

There was no significant difference in predator-density between emigration and non-emigration ponds, both overall (Wilcoxon-rank-sum-test, W = 242, p = 0.77); as well as when different sampling events were compared (sampling 1: W = 5, p = 0.11; sampling 2: W = 31, p = 0.88; sampling 3: W = 51, p = 0.27). In total, dragonfly larvae were the most abundant predators (more than 71 % of all predators, n = 244), followed by *Ichthyosaura alpestris* (n = 44) and Dyctisidae (n = 21; predominantly larvae).

*Rana temporaria* tadpole-density (tadpoles per m^3^: t/m^3^) decreased significantly with time (mean ± SD, sampling 1: 3984.48 t/m^3^±6636.64; sampling 2: 373.25 t/m^3^±699.63; sampling 3: 125.94 t/m^3^±221.38; Friedman-Test, χ^2^ = 9.8, df = 2, p = 0.007). Between first and second sampling, tadpole-density dropped by factor ten (Wilcoxon-signed-rank-test, V = 49, p = 0.032). Although mean tadpole-density also decreased between second and third sampling, this difference was not significant (V = 52, p>0.1, see Table 1).

### Survival to metamorphosis

Survival rate varied considerably between study ponds. Using visual inspection and box-sampling, tadpoles could be found in only 11 of 18 breeding ponds. For these ponds, survival rate to metamorphosis averaged at 1.58 %±1.57 (range: 0.23–5.24%; n = 11; Table 1). Although the number of clutches varied between 1 and 190 (Table 1), there was no significant correlation between survival rate and clutch number (Spearman-rank correlation, S = 627.82, rho = 0.352 p = 0.15, n = 18).

### Are there environmental differences between emigration and non-emigration ponds?

**Figure 2:**
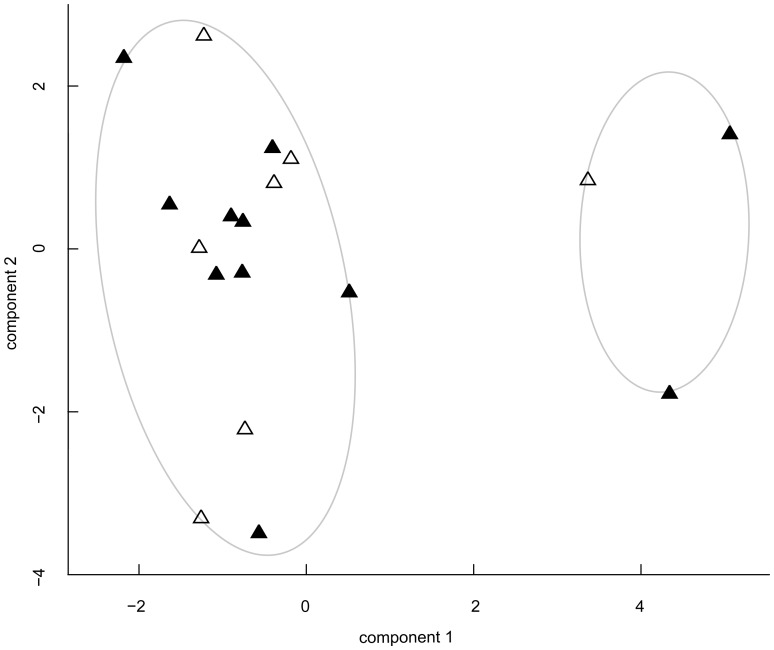
Study ponds assigned according to the results of the k-means clustering. Ponds are plotted in the first two principal components using 16 abiotic habitat characteristics, explaining 40.53 % of data variance. Ponds with emigrating *Rana temporaria* metamorphs are indicated by filled, non-emigration ponds by open triangles. Loadings of the components are given in Table S3.

In order to identify potential differences in abiotic parameters between emigration and non-emigration ponds, we predefined the number of groups (two groups) in k-means clustering. These two groups contained three (WB04, WB07 and FS06) and 15 ponds respectively and explained 82.6 % of the variance in environmental parameters [total within sum of squares/total sum of squares], but failed to group ponds according to emigration (11 ponds) and non-emigration ponds (7 ponds, Figure 2, Table S3), as did the hierarchical clustering (Figure S1).

Random forest analysis likewise was not able to adequately classify emigration and non-emigration ponds (mtry = 2, ntree = 10000) – with 38.9 % error rate (‘out of bag estimation’) the correct assignment to a group was rather poor: for emigration, seven of 11 ponds were classified correctly (error rate 18.2 %), but only two of seven non-emigration ponds were classified correctly (error rate 71.4 %). The most important variables which contributed to a correct classification were water depth, structuring vegetation and temperature.

Finally, principal component analysis was used to reduce the number of abiotic parameters. The first five axes explained 72.3 % of the total variance. These axes were subsequently used in logistic regression with quasibinomial error distribution to explain survival rate in the 18 ponds. However, the full model failed to validate an overall effect of the combined factors when compared to the null model (model with no explanatory variables, p = 0.67, see Table S4) and was therefore not further used.

### Metamorphic traits

Between 13 June and 5 October 2007, a total of 7348 juveniles emigrated from 11 ponds (Table 1). Pond specific average development time, defined as time from spawning date to metamorphosis, ranged between 95.7±16.4 and 124.2±16.7 days (Table 1) and differed significantly between ponds (Kruskal-Wallis-χ^2^ = 828.96, df = 10, p<0.001). The first juveniles emigrated after 79 days, the last ones after 196 days. Furthermore, the emigration period (time between the first and the last metamorphing juvenile in the respective pond) highly differed between ponds, ranging from 59 to 114 days.

**Figure 3:**
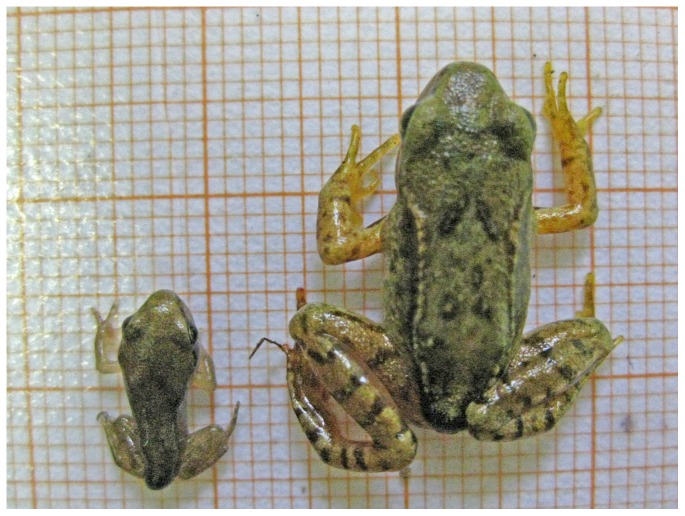
*Rana temporaria* metamorphs from two different ponds in the study area. Although being in the same developmental stage and emigrating at the same date, the differences in size, proportion and vitality are obvious. Individual left: 10.2 mm, 0.09 g, right: 17.2 mm, 0.56 g, 23 July 2009.

Metamorphic size varied profoundly among ponds (Kruskal-Wallis-χ^2^ = 3531.69, df = 10, p<0.001). The smallest juveniles emigrated from pond AW03, exhibiting sizes of 11.3 mm±0.7, thus being more than 4 mm (25.6%) smaller than the largest metamorphs (pond WG02, average size 15.6 mm±1.2). Among ponds minimum sizes varied between 9.0 mm (AW09) and 12.6 mm (WG02), maximum sizes ranged from 14.4 mm (AW03) to 21.4 mm (WG07). Within ponds, coefficient of variation (CV) in size was between 6.0 and 12.3%. As expected, metamorphic mass was highly correlated with size (Spearman-rank correlation, p<0.001, rho = 0.937), but variation in mass was even higher than in size. Accordingly, we could detect significant differences in body mass among ponds (Kruskal-Wallis-χ^2^ = 3682.51, df = 10, p<0.001). The CV of mass was between 15.2 and 39.3% at metamorphosis. Juveniles of pond AW03 had lowest body mass (0.14 g±0.02). Highest masses were found in juveniles from pond WG02 (0.39 g±0.09). The lightest individual weighed just 0.07 g (AW09), factor ten less than the biggest metamorph (0.70 g; WG07; Table 1, see also Figure 3).

### Do biotic and/or abiotic factors explain metamorphic trait differences?

We fitted multiple linear regression models to identify environmental parameters influencing differences of the median in metamorphic traits between the ponds. In order to avoid an overfit of the model we reduced the number of explanatory variables [53]. A PCA with all biotic and abiotic parameters was performed and the first three axes, explaining 59% of variance, were included in the respective models. However, linear models failed to proof explanatory power when compared to the respective null model, and therefore were not further used (ANOVA: F_size_ =  1.2657, p = 0.36, F_mass_ =  1.5879, p = 0.28, F_development time_ =  0.2185, p = 0.88).

### Do environmental factors influence emigration patterns?

**Figure 4:**
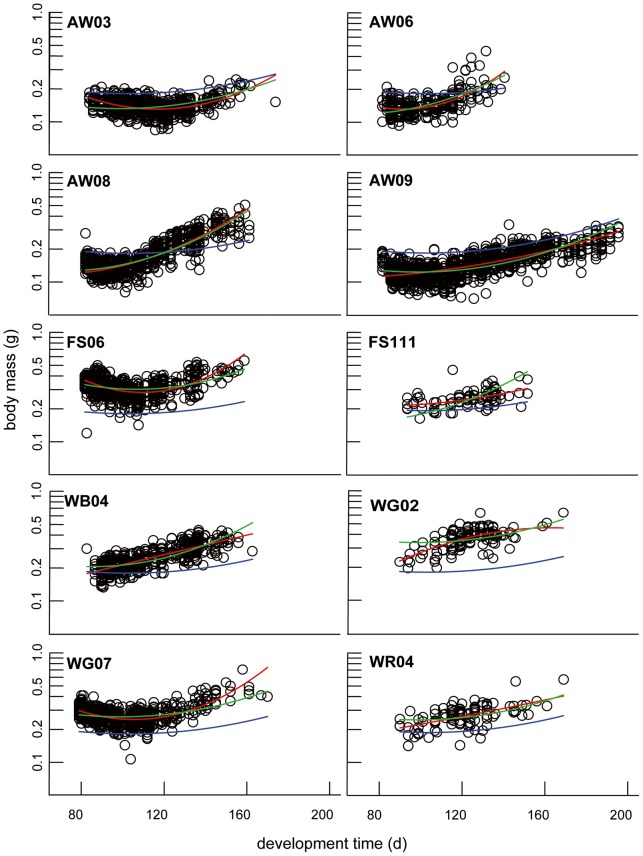
Emigration patterns of *Rana temporaria* metamorphs. Linear regression models describing *R. temporaria* metamorphic mass in relation to development time for the individual ponds were fitted in three approaches: first, all metamorphs irrespective of their emigration pond, resulting in an average regression for the studied population [blue line, log(mass) ∼ −0.83650 + (−0.01697× development-time) + (0.00008× development-time^2^)]. Second, regressions were calculated for each pond independently (red line, for exact description for regression parameters see Table S5). Finally, best linear mixed model according to model selection was used describing metamorphic mass over development time with the assessed environmental parameters (green line: log(mass) ∼ structuring-vegetation × development-time + inflow × development-time^2^, ‘pond’ was used as random factor (see Table 2).

We observed profound differences between ponds when metamorphic mass of juveniles was fitted against the respective development time of each individual (Figure 4). Therefore, we used quadratic regression models to describe and match the shape of the observed patterns (Figure 4, Table S5). The regressions were fitted according to: log(mass) ∼intercept + x × development-time + z × development-time^2^. We used two different approaches. First, we fitted a quadratic regression using all emigrated juveniles irrespective of their pond association, resulting in an overall average regression (blue line in Figure 4) for the whole population. Second, in order to compare the overall mean of the population with individual ponds, we fitted a second quadratic regression with the respective juveniles for every pond individually (red lines), resulting in a better match of regression for the individual ponds. In order to ascertain a potential correlation of emigration patterns and environmental parameters, we used the slopes of the individual regressions as a surrogate for curve shapes. Based on the confidence intervals of the slopes, we defined two groups of ponds: group A: WR04, WG02, WB04, AW09, AW08 and group B: WG07, FS06, AW06, AW03 (FS111 was excluded to accentuate the difference, Figure S2). Subsequently, we checked for differences in habitat characteristics between these two groups using Mann-Whitney-U tests. However, we could not detect any significant differences in the environmental parameters of these two groups (Table S6).

In order to identify habitat characteristics best describing the shape of the observed emigration patterns, we applied model selection with a linear mixed model using pond as random factor. We assigned the 16 pond parameters as well as average predator- and tadpole-density as coefficients for slope (x) and bend (z) of the quadratic regression curves in all possible combinations. The different models were then ranked according their AICc, which estimates the lack of fit of the model to the real data and penalizes models for greater complexity [47]. The ‘best’ model had the lowest AICc. In our case the amount of structuring vegetation in the ponds (x) and presence of an inflow of water from nearby streams (z) showed far the best result (lowest AICc: −4118.14, ΔAICc to 2nd ranked model = 85.13, green line, Table 2). Other combinations of parameters showed essentially no empirical support, since the difference to the AICc of the best model was >10 [46]. Nevertheless, even the best model (green line) did not match the curve shape in all cases (see Figure 4).

**Table 2:** Most important parameters (see Table S1) describing the pattern of *Rana temporaria* metamorphosis mass in study ponds.

environmental parameter					
x	Z	log-likelihood	AICc	ΔAICc	Akaike mass
structuring vegetation	Inflow	2068.1	−4118	0	1.0
canopy openness	structuring wood	2025.5	−4033	85	0.0
duckweed	Inflow	2020.1	−4022	96	0.0
inflow	structuring vegetation	2015.3	−4013	106	0.0
structuring wood	canopy openness	1992.1	−3966	152	0.0
structuring vegetation	shore vegetation	1980.1	−3942	176	0.0
ammonium	shore vegetation	1979.1	−3940	178	0.0
inflow	duckweed	1974.5	−3931	187	0.0

Emigration mass in study ponds (n = 10, Figure 4) is described according to: log(mass) ∼x × development-time +z × development-time^2^. Given are the parameter combinations for the coefficients of slope (x) and bend (z) of the curve in the linear mixed model, which showed the lowest AICc (Akaike Information Criterion for finite sample sizes) after model selection (i.e. ΔAICc = AICc_i_−AICc_min_, <200). ‘Pond’ was used as random factor. Akaike mass  =  normalized relative likelihood of the model given the data.

### Is there a pond signal in metamorphic traits, using data of multiple years?

**Figure 5:**
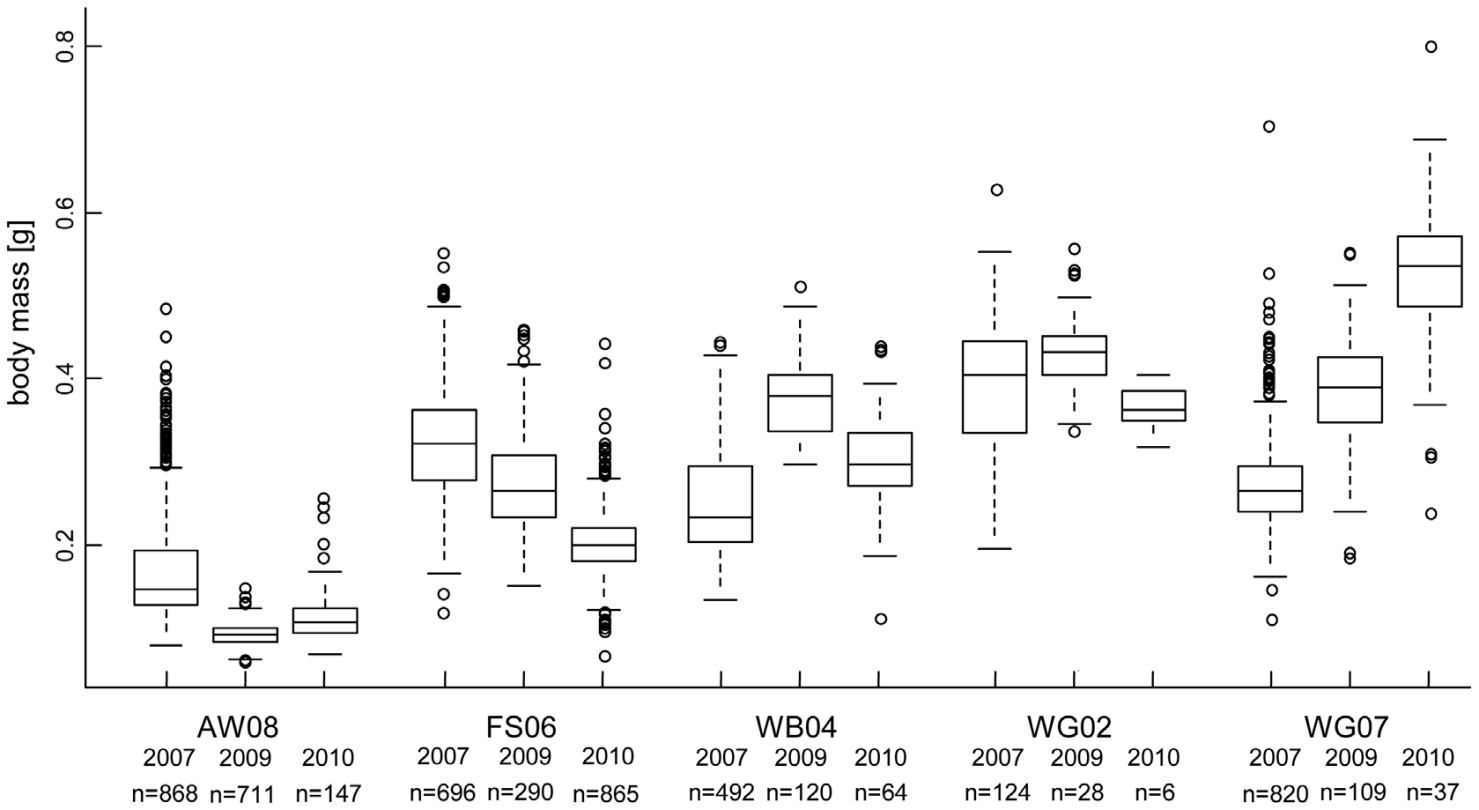
Metamorphic mass *of Rana temporaria* for five ponds in the years 2007, 2009 and 2010. For values of size and development time, see Table S7.

When we compared the metamorphic traits of those five ponds where data for three years were available, no pattern could be observed (Figure 5, Table S7). In an analysis of variance (ANOVA), both main effects (pond, year) as well as the interaction of these factors (pond X year) were significant for development time, metamorphic mass and size (Table 3).

**Table 3:** ANOVA for mass, size, and development time of metamorphosing *Rana temporaria*.

Response	Factor	df	F-ratio	*P*-value
development time	Pond	4	72.58	<0.001
	Year	2	27.98	<0.001
	pond×year	8	25.92	<0.001
Size	Pond	4	2594.23	<0.001
	Year	2	310.38	<0.001
	pond×year	8	319.31	<0.001
mass	Pond	4	2401.41	<0.001
	Year	2	503.93	<0.001
	pond×year	8	446.57	<0.001
	Residual	5362		

Given are the data for five ponds and three years. df  =  degrees of freedom.

Survival decreased with years in the five monitored ponds (2007: 2.80 %, 2009: 1.34 %, 2010: 0.51 %) and differed significantly both between ponds and years (Friedman-test, χ^2^_pond_ = 9.87, df = 4, p = 0.043; χ^2^_year_ = 8.4, df = 2, p = 0.015; Table 1, Table S7). Thus, pond specific characteristics are not likely to cause the observed differences in metamorphic traits.

## Discussion

In this study, we aimed to assess the influence of the environment on metamorphic traits (developmental time, size and mass at metamorphosis) in *Rana temporaria* tadpoles. The investigated population exhibits a strong fidelity in oviposition site use, using particular ponds and avoiding others, which indicates a selection of breeding habitats [31]. Therefore, we assumed that adults choose an environment suitable for larval development. Yet, in this study, tadpoles failed to survive until metamorphosis in seven out of 18 ponds. Although amphibians are known to select oviposition sites according to environmental factors (e.g. [54], [55]), we could not detect any distinct differences between ponds where tadpoles reached or failed to reach metamorphosis. Furthermore, in ponds with emigrating juveniles, we detected unexpectedly high plasticity in size, mass and development time of metamorphs, both between and within ponds.

### Which factors cause variation in metamorphic traits within ponds?

The maximum coefficient of variation (CV) was 0.39 in metamorphic mass within single ponds and average mass differed up to factor 2.84 between ponds. This high divergence of larval developmental performance is likely to shape also frogs' performance in later life stages, due to its effects on mobility, survival and fecundity [56]–[58]. We are only aware of few amphibian studies reporting variation of metamorphic traits for entire cohorts (salamanders:[14]–[16], [59], [60], frogs: [11], [61], [62]). Berven [11] reports variances between 14.2 and 18.1 mm in *Lithobates sylvaticus* metamorphic size, resulting in CVs of 0.07–0.08 in two neighbouring ponds. These CV values are in the range of our study (0.06–0.12), however, without the extremes reported herein (min: 9 mm, max: 21.4 mm). Here, the variance of metamorphic mass was even more pronounced, being one order of magnitude between individuals.

The observed differences in metamorphic traits suggest differences in the developmental habitat, however, we could not detect a correlation between the metamorphic timing and mass, and the factors, which are commonly assumed to influence amphibian development most (and already proved their impact in laboratory and mesocosm studies: predation ([63]–[65] summarized in [21], [66]), intraspecific competition [10], [18], [67], [68], temperature [69], [70], desiccation risk [4], [25], [71] or food availability [26], [72]. Only two of all factors assessed correlated with metamorphic mass. Nitrate was associated negatively, however, concentrations varied between 0 and 2 mg/l, and thus were far below the levels to expect any adverse effects [73], [74]. Correlation of duckweed cover (a plant forming dense floating carpets) was positive. Duckweed might not influence tadpole performance directly, but affects parameters such as solar radiation, gas exchange and temperature. Additionally, it indicates ponds with prolonged hydroperiods (the plant being absent in ponds which dry up). However, the correlation of duckweed and metamorphic mass in our study remains ambiguous: although duckweed covered 0–70 % of the water surface in emigration ponds, the distribution was highly skewed towards zero: eight ponds showed no duckweed cover, the remaining three ponds showed values of 10, 45 and 70 % respectively.

### Emigration patterns

In addition to the extreme trait variances, we observed very different curve shapes when metamorphic mass was plotted against development time. These ranged in different ponds from linear to U-shaped to asymptotic. Usually, there is a positive correlation between metamorphic mass and development time in amphibians [24], [75]. When applying linear regression, we found both positive and negative relationships; however, these regressions poorly met the pattern of the data. When using environmental parameters to describe the curve shape in a quadratic regression, model selection favoured structuring vegetation and the presence of inflow over other factors, such as predation or competition, to describe the progression of the curve. Although we do not deny a potential impact of these parameters, this result might be ambiguous. The respective parameter data included a high number of zero values, e.g. in eight of eleven emigration ponds no structuring vegetation existed. Since patterns of metamorphosis mass highly differed between ponds, we assumed that different mechanisms may have acted in different ponds. For instance, intra-population variation caused by genetic, stochastic, maternal or environmental (microhabitat) effects [76]–[78] could give some tadpoles head-start advantages. This could have led to an early emigration of these few advantageous tadpoles with comparatively high mass, whereas high competition conditions results in a majority of tadpoles metamorphosing with lower mass, followed by a subsequent competitive release for the remaining tadpoles and resulting in a U-shaped emigration pattern. However, there was no such correlation between coefficients of the emigration curves and predator- or tadpole-density.

### Why is there no correlation?

The question arises, why none of the multiple factors we examined showed a significant correlation to metamorphic traits or cohort variance. First, variance of environmental parameters in the natural habitats might be continuous and smaller than in experimental designs, which often test extremes. Therefore, the contrast in the individual responses to these environments might be less pronounced and difficult to detect. In our study this might apply to some abiotic factors, but not to the biotic ones. Tadpole- (up to factor 700) and predator- (0–540 predators/m^3^) densities varied widely between ponds, resulting in pronounced differences in competition and mortality risk. Thus, it is unlikely that the lack of detectable effects of competition and predation on metamorphic traits was due to an insufficiently strong gradient.

Second, although various factors might be highly influential on metamorphic traits, these factors might be inter- and counteracting. For instance, predation can relax intraspecific competition indirectly by thinning and consequently improve the developmental conditions for the survivors [75], [79], [80]. This could lead to different outcomes depending on the magnitude of the respective stressor. In general, the effect of thinning should be highest when competition is high [81]. In our study, although densities were high in some ponds, there was no correlation neither of predator-density and tadpole mortality between the sampling events, nor tadpole-density, or any metamorphic traits.

Third, the reaction to different environmental stressors can have different effects, depending on the time when the stressor is presented during development (summarized in [82]) and there is growing evidence that stressful conditions, such as high tadpole densities, experienced in early development influence metamorphic traits as well as fitness of later ontogenetic stages [58], [83]. Thus, complex interactions between environmental stressors and the state in growth and development of the individuals may hamper identification of most important parameters under natural conditions.

Fourth, comparisons between natural habitats might be potentially difficult, if environmental factors vary much on small spatial scales. Such ‘environmental noise’ might overwrite effects despite strong cues of the assessed factors, e.g. in a full pond experiment Loman [84] detected a density effect on the development of *R. temporaria* larvae, but registered that this variation in metamorphic traits was lower than the variation caused by pond identity and years. Since we focused on ponds spread over small geographic scale and located in a temperate forest environment with little forestry activity, inter-annual variation of environmental factors was likely to act equally on all our study ponds. Thus, if the ponds' environment would be responsible for pond specific pattern in metamorphic traits, inter-annual differences should result in similar directions of larval responses in the monitored ponds. However, this was not the case, in contrast, metamorphic traits showed very different patterns not only between ponds but also between years.

We thus interpret the heterogeneity of our results as complex tadpole-environment interactions, acting on an individual level with the direction and magnitude of individual responses to environmental cues potentially being very different. These differences might be more pronounced in natural set ups with many varying parameters than in experiments.

To unravel the potential range of responses within populations, studies like ours are needed to validate the importance of single factors identified within experiments in natural environments. An important outcome of this study is the magnitude of intra-population variance of metamorphic traits. Variation in size and development is a widespread phenomenon of populations, which strongly influences population dynamics (reviewed in [85]), or strength and direction of natural selection [86]. However, the great majority of studies focus on trait mean values of a population, produced by environmental variability [87], but studies as ours hint also towards genetic background shaping considerably response potential of individuals. Profound differences in life history traits due to local genetic differentiation have already be shown, also over short geographical scales, for *R. temporaria* [28], [88] and could also be present in the studied population. The ecological effects of such intraspecific variation in traits can be large [89], [90]. As selection and adaptation processes act on individuals, it is vital to assess variability of reaction norms on the individual level to gain knowledge about the ability of natural systems to cope with environmental change [6], [89]. Transfer-experiments within populations could give important cues on plastic responses caused by environmental factors and additionally reveal potential adaptation processes on very local scale. In our opinion, this study emphasizes the need of studies on natural populations to reassess ecological relationships and to identify selective forces and genetic differentiation within populations in the field.

## Supporting Information

Table S1 **Summary and description of environmental parameters.**
(PDF)

Table S2 **Correlation results of environmental parameter with metamorphic traits.**
(PDF)

Table S3 **Component loadings used in visualization of the k-means clustering.**
(PDF)

Table S4 **Summary of GLM describing survival of** ***Rana temporaria*** **in relation to environmental principal components.**
(PDF)

Table S5 **Summary of regression of the emigration pattern of** ***Rana temporaria*** **juveniles.**
(PDF)

Table S6 **Summary statistics for regression slopes of emigration patterns.**
(PDF)

Table S7 **Summary of metamorphic traits of** ***Rana temporaria*** **for 2009 & 2010.**
(PDF)

Figure S1 **Hie rarchical cluster of environmental parameters.**
(PDF)

Figure S2 **Confidence intervals of coefficients of regression models of emigration patterns.**
(PDF)
