# Supplementary material for: Giants, Dwarfs and the Environment – Metamorphic Trait Plasticity in the Common Frog
Source: PLoS One. 2014 Mar 5;9(3):e89982. doi: 10.1371/journal.pone.0089982 (PMC3943853; doi:10.1371/journal.pone.0089982)
Supplement: Table S1 — Summary and description of environmental parameters. (PDF) [file pone.0089982.s001.pdf]

Table S1. Summary of parameter variability and definition at 18 breeding ponds of *Rana temporaria* in northern Bavaria, Germany. Given is the mean and standard deviation (mean ± sd), as well as minimum and maximum values or frequency of ponds in the respective categories. Water temperature (n = 13) could not be evaluated for every pond, missing values for ponds AC01, AW04, RS04Rinne, RS08, WB07 were substituted by the overall temperature mean.

**variable**	**mean ± sd**	**min**	**max**	**description**
canopy openness [%]	17.90 ± 4.76	10.56	32.98	canopy openness calculated using Gap Light Analyzer Version 2.0 (Frazer et al. 1999)
duckweed cover [%]	11.44 ± 20.42	0.00	70.00	% of water surface covered by duckweed, *Lemna* sp.
structuring vegetation[%]	11.33 ± 18.10	0.00	60.00	% of water surface structured by plants
shore vegetation [%]	43.00 ± 33.45	0.00	100.00	% of surface covered by vegetation within a 0.5m shore line
structuring wood [%]	22.61 ± 15.40	7.00	60.00	% water surface structured by dead wood, logs etc
water depth (incl.sediment)[m]	0.28 ± 0.13	0.07	0.57	mean water depth calculated from 13 points (centre, as well as 0.1m, 0.5m,1m in all cardinal directions from centre)
volume [m^3]	5.17 ± 7.11	0.06	27.49	volume based on volume= 0.5 length * 0.5 breadth * water depth in centre * 2/3 π.
water temperature [°C]	14.35 ± 0.56	13.39	15.27	mean water temperature calculated from continuous recordings 0.1m below water surface using data loggers (IButtons, Maxim ±0.5°C, 2h interval) between April 14 and August 21
variation water depth	19.97 ± 24.38	3.81	101.39	variation coefficient calculated of seven measurements of water depth during study period
pH	6.93 ± 0.52	5.88	7.99	pH-value below surface (± 0.1), mean of two samplings (May 21; June 19), measured in 0.5m distance to shoreline using Water Tester HI98204 HANNA Instruments, precision 0,1 pH, Kehl am Rhein, Germany)
nitrate (NO_3) [mg/l]	0.44 ± 0.37	0.00	1.00	nitrate content mean of two samplings (May 21; June 19), for each sampling, three water samples were collected at pond bottom in 0.5m distance to shoreline using Visocolor© Eco nitrate (4 - 120 mg/l), Macherey-Nagel, Düren, Germany.
ammonium (NH_4) [mg/l]	1.24 ± 0.83	0.38	3.00	ammonium content mean of two samplings (May 21; June 19), for each sampling, three water samples were collected at pond bottom in 50cm distance to shore line using Visocolor© Eco ammonium 15 (0,2 - 3 mg/l), Macherey-Nagel, Düren, Germany
phosphate (PO_4) [mg/l]	0.49 ± 0.17	0.25	0.90	phosphate content mean of two samplings (May 21; June 19),for each sampling, three water samples were collected at pond bottom in 0.5m distance to shore line using Visocolor© Eco phosphate (0,2 - 5 mg/l), Macherey-Nagel, Düren, Germany
underwater vegetation [%]*	2.22 ± 6.47	0.00	20.00	% of water surface structured by underwater vegetation
turbidity [category 1-4]	1.94	1.11		visual inspection of turbidity [1=clear, 2=lightly turbid, 3= turbid, 4= highly turbid]
inflow [0;1]	absence: 12	presence: 6		absence or presence of inflow [0;1]
pond bottom [0;1]	absence:11	presence: 7		leaf litter or mud on pond bottom [0;1]
sapropel [0;1]*	absence: 0	presence:18		absence or presence of sapropel in pond [0;1]

*parameters were excluded from further analyses due to lack of variance in parameter values

Frazer GW, CanhamCD, Sallaway P, Marinakis D (1999) Gap Light Analyzer. -Simon Fraser University Burnaby, British Columbia, Canada. Institute of EcosystemStudies, Milbrook, New York, USA.
