# Supplementary material for: Giants, Dwarfs and the Environment – Metamorphic Trait Plasticity in the Common Frog
Source: PLoS One. 2014 Mar 5;9(3):e89982. doi: 10.1371/journal.pone.0089982 (PMC3943853; doi:10.1371/journal.pone.0089982)
Supplement: Table S2 — Correlation results of environmental parameter with metamorphic traits. (PDF) [file pone.0089982.s002.pdf]

Table S2. Spearman rank correlation of assessed environmental parameter with metamorphic traits of *Rana temporaria*. Given are the environmental parameters and the test statistic (correlation coefficient R and P-value

**variable**	**survival rate**		**SVL**		**body mass**		**development time**	
	***P*-value**	**R**	***P*-value**	**R**	***P*-value**	**R**	***P*-value**	**R**
canopy openness	0.73	-0.12	0.79	-0.09	0.71	-0.13	0.89	-0.05
duckweed	0.63	-0.16	**0.01**	**0.72**	**0.02**	**0.67**	0.89	-0.05
structuring vegetation	0.91	-0.04	0.33	0.32	0.44	0.26	0.22	-0.40
shore vegetation	0.85	0.06	0.47	0.25	0.65	0.15	0.83	-0.07
turbidity	0.90	0.04	0.12	0.49	0.14	0.47	0.32	0.33
structuring wood	0.76	-0.11	0.75	0.11	0.79	0.09	0.63	0.17
inflow	0.15	-0.46	0.87	0.06	0.87	-0.06	0.49	-0.23
pond bottom	0.22	-0.40	0.61	0.17	0.74	0.12	1.00	0.00
water-depth	0.57	0.19	0.35	0.31	0.32	0.33	0.11	-0.51
volume	0.47	0.25	0.07	0.57	0.13	0.49	0.42	-0.27
water temperature	0.38	0.31	0.19	-0.45	0.21	-0.43	0.17	0.47
variation water depth	0.54	-0.21	0.34	-0.32	0.39	-0.29	0.63	-0.16
pH	0.50	-0.23	0.18	0.44	0.17	0.45	0.83	-0.07
nitrate	0.72	0.12	**0.04**	**-0.62**	0.08	-0.56	0.33	-0.32
ammonium	0.20	-0.42	0.52	0.22	0.54	0.21	0.16	0.45
phosphate	0.62	0.17	0.99	0.00	0.95	0.02	0.99	0.00
predator density	0.86	0.06	0.98	-0.01	0.87	-0.05	0.13	0.49
larval density	1.00	0.00	0.52	-0.22	0.33	-0.33	0.47	-0.25
