# Supplementary material for: Giants, Dwarfs and the Environment – Metamorphic Trait Plasticity in the Common Frog
Source: PLoS One. 2014 Mar 5;9(3):e89982. doi: 10.1371/journal.pone.0089982 (PMC3943853; doi:10.1371/journal.pone.0089982)
Supplement: Table S3 — Component loadings used in visualization of the k-means clustering. (PDF) [file pone.0089982.s003.pdf]

Table S3. Loadings of the first two components of the PCA-axes used in visualization of the result of the k-means clustering (Figure 2). For further information see methods and Figure 2.

	loading	
variable	component 1	component 2
canopy openness	0.270	0.300
duckweed cover	0.256	-0.382
structuring vegetation	0.303	0.100
shore vegetation	0.371	0.002
turbidity	-0.149	-0.267
structuring wood	0.082	-0.264
inflow	0.393	0.096
pond bottom	0.107	0.263
water depth (incl.sediment)	0.092	-0.145
volume	0.420	-0.055
variation water depth	-0.276	0.290
pH	-0.074	-0.040
nitrate (NO_3)	-0.304	0.350
ammonium (NH_4)	-0.084	-0.421
phosphate (PO_4)	-0.162	-0.311
water temperature	-0.211	-0.168
