# Supplementary material for: Giants, Dwarfs and the Environment – Metamorphic Trait Plasticity in the Common Frog
Source: PLoS One. 2014 Mar 5;9(3):e89982. doi: 10.1371/journal.pone.0089982 (PMC3943853; doi:10.1371/journal.pone.0089982)
Supplement: Table S4 — Summary of GLM describing survival of Rana temporaria in relation to environmental principal components. (PDF) [file pone.0089982.s004.pdf]

Table S4. Generalized linear model (GLM with quasibinomial error distribution) of the proportional survival of *Rana temporaria* metamorphs in the study ponds, given is the result of the χ^{2}-test resulting in deviance, number of degrees of freedom (df_1), residual degrees of freedom (df_2) and significance for each PCA-axis.

**parameter**	**deviance**	**df_1**	**df_2**	***P*-value**
PC5	0.11	1	12	0.9913
PC4	62.80	1	13	0.7973
PC3	3.93	1	14	0.9488
PC2	536.12	1	15	0.4531
PC1	2342.81	1	16	0.1168
Residual error	9424.20			
Total	12370.00			
