# Supplementary material for: Giants, Dwarfs and the Environment – Metamorphic Trait Plasticity in the Common Frog
Source: PLoS One. 2014 Mar 5;9(3):e89982. doi: 10.1371/journal.pone.0089982 (PMC3943853; doi:10.1371/journal.pone.0089982)
Supplement: Table S5 — Summary of regression of the emigration pattern of Rana temporaria juveniles. (PDF) [file pone.0089982.s005.pdf]

Table S5. Coefficients of the regression used to describe *Rana temporaria* metamorphic weight in relation to development time according to the equation: log(weight)~ intercept +x×development-time + z×development-time^2.

Pond #	intercept	x	z
AW03	0.96726	-0.05147	0.00022
AW06	0.75044	-0.06075	0.00033
AW08	-1.46925	-0.01957	0.00015
AW09	-2.16100	-0.00407	0.00004
FS06	2.71601	-0.07256	0.00033
FS111	-1.19156	-0.01081	0.00007
WB04	-2.63307	0.01129	0.00000
WG02	-4.46331	0.04608	-0.00014
WG07	1.52047	-0.05555	0.00026
WR04	-2.22523	0.00582	0.00001
