# Supplementary material for: Giants, Dwarfs and the Environment – Metamorphic Trait Plasticity in the Common Frog
Source: PLoS One. 2014 Mar 5;9(3):e89982. doi: 10.1371/journal.pone.0089982 (PMC3943853; doi:10.1371/journal.pone.0089982)
Supplement: Table S6 — Summary statistics for regression slopes of emigration patterns. (PDF) [file pone.0089982.s006.pdf]

Table S6. Results of the comparison of pond characteristics between the two groups of ponds differing in slope of the observed individual emigration pattern (metamorphic weight over development time) of *Rana temporaria*. See methods and results for details. Given is the test statistic W and *P*-values of the Mann-Whitney-U test.

**variable**	**W**	***P*-value**
canopy openness	3	0.111
duckweed	9.5	1.000
structuring vegetation	9.5	1.000
shore vegetation	6	0.413
tubridity	9.5	1.000
structuring wood	7	0.537
inflow	11	0.888
pond bottom	11	0.888
water-depth	15	0.286
volume	9	0.905
pH	7	0.556
nitrate	16	0.161
ammonium	6.5	0.461
phosphate	16	0.174
water temperature	4	0.191
variation water depth	15	0.286
predator-density	2.5	0.085
tadpole-density	12	0.730
