# Supplementary material for: Giants, Dwarfs and the Environment – Metamorphic Trait Plasticity in the Common Frog
Source: PLoS One. 2014 Mar 5;9(3):e89982. doi: 10.1371/journal.pone.0089982 (PMC3943853; doi:10.1371/journal.pone.0089982)
Supplement: Table S7 — Summary of metamorphic traits of Rana temporaria for 2009 & 2010. (PDF) [file pone.0089982.s007.pdf]

Table S7. Metamorphic traits of *Rana temporaria* in the years 2009 and 2010. Given is the mean and standard deviation (SD) or coefficient of variance (CV), as well as the minimum and maximum size and weight of the measured individuals. Survival rate was based on 1117 eggs/clutch.

			**metamorphs**	**survival**	**size**				**weight**				**development time**			
**pond**	**year**	**clutches**	**(total/meas)**	**[%]**	**mean±SD**	**min**	**max**	**CV**	**mean±SD**	**min**	**max**	**CV**	**mean±SD**	**min**	**max**	**CV**
AW08	2009	60	2310 / 711	3.45	10.3 ± 0.6	8.7	11.8	0.06	0.09 ± 0.01	0.06	0.15	0.15	109.6 ± 21.5	74	159	0.20
AW08	2010	120	212 / 147	0.16	10.9 ± 0.9	8.9	14.2	0.08	0.11 ± 0.03	0.07	0.26	0.27	113.7 ± 9.7	99	144	0.08
FS06	2009	33	812 / 290	2.20	14.4 ± 1.0	11.7	17.3	0.07	0.27 ± 0.06	0.15	0.46	0.21	99.7 ± 11.2	83	140	0.11
FS06	2010	86	2044 / 865	2.13	13.1 ± 0.9	8.9	16.6	0.07	0.20 ± 0.04	0.06	0.44	0.19	106.7 ± 11.1	88	151	0.10
WB04	2009	139	208 / 120	0.13	15.6 ± 0.7	14.1	17.2	0.04	0.38 ± 0.05	0.30	0.52	0.12	97.5 ± 4.0	91	106	0.04
WB04	2010	382	101 / 64	0.02	14.4 ± 1.0	10.7	16.8	0.07	0.30 ± 0.06	0.11	0.44	0.19	100.8 ± 4.5	95	112	0.04
WG02	2009	14	44 / 28	0.28	16.4 ± 0.9	14.5	18.6	0.05	0.43 ± 0.06	0.34	0.56	0.13	96.9 ± 4.5	90	107	0.05
WG02	2010	11	6 / 6	0.05	15.2 ± 0.8	13.9	16.4	0.05	0.36 ± 0.03	0.32	0.40	0.08	98.3 ± 0.8	98	100	0.01
WG07	2009	17	118 / 109	0.62	15.6 ± 1.0	12.6	17.5	0.06	0.39 ± 0.07	0.18	0.55	0.18	101.2 ± 9.2	88	128	0.09
WG07	2010	25	54 / 37	0.19	16.9 ± 1.1	14.0	19.6	0.07	0.52 ±0.11	0.22	0.78	0.21	109.3 ± 5.5	98	122	0.05

metamorphs (total/meas) = number of metamorphs (emigrated/measured)
