# Supplementary material for: Giants, Dwarfs and the Environment – Metamorphic Trait Plasticity in the Common Frog
Source: PLoS One. 2014 Mar 5;9(3):e89982. doi: 10.1371/journal.pone.0089982 (PMC3943853; doi:10.1371/journal.pone.0089982)
Supplement: Figure S1 — Hie rarchical cluster of environmental parameters. (PDF) [file pone.0089982.s008.pdf]

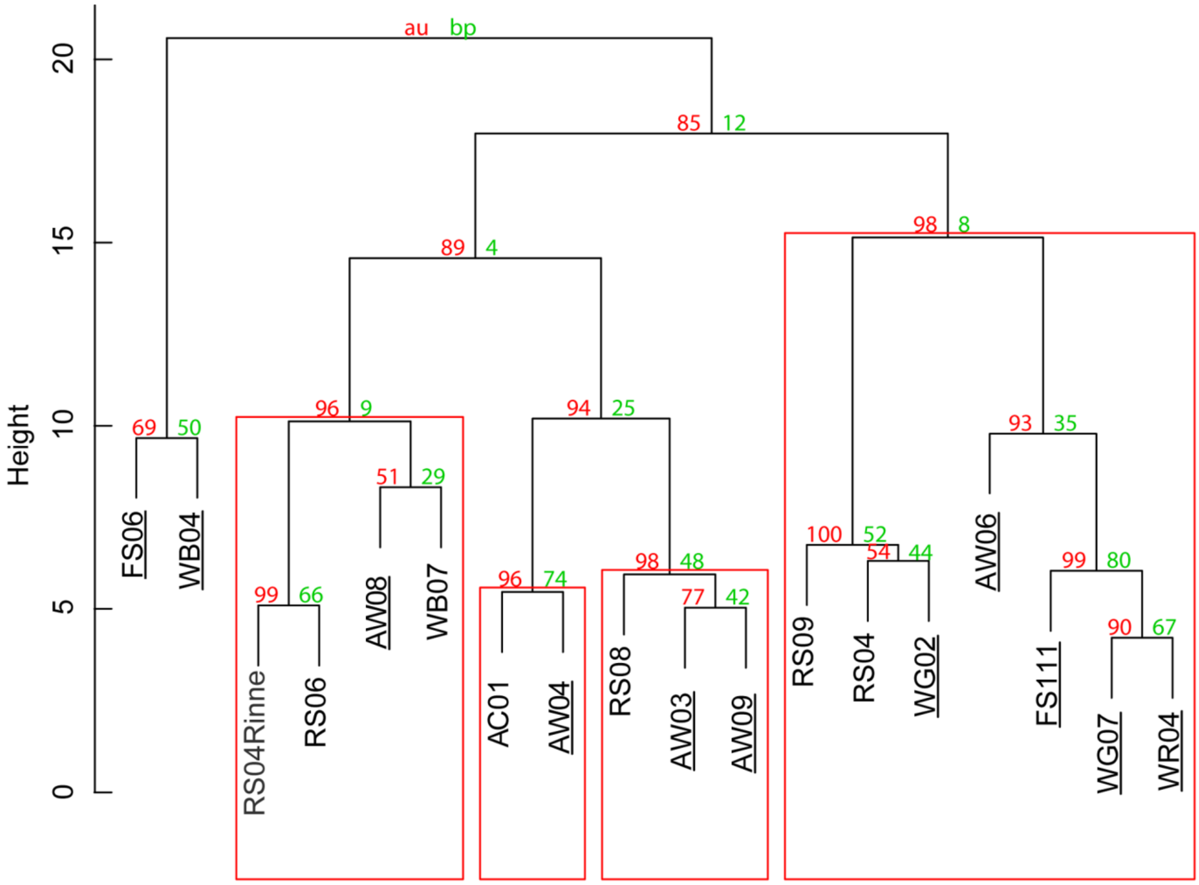

Figure S1. Study ponds grouped by scaled abiotic habitat parameters based on Ward's clustering of Euclidean dissimilarity matrix and multiscale bootstrap resampling (n = 1000). Given are au (approximately unbiased, Shimodaira 2002) and bp (bootstrap probability) p-values for the respective clusters. Clusters which are strongly supported by the data (au>0.95) are highlighted by rectangles. Emigration ponds are underlined. There is no pattern in environmental characteristics explaining failure of emigration of *Rana temporaria* in some ponds.

Shimodaira H (2002) An approximately unbiased test of phylogenetic tree selection. Systematic Biology 51: 492–508.
