# Supplementary material for: Giants, Dwarfs and the Environment – Metamorphic Trait Plasticity in the Common Frog
Source: PLoS One. 2014 Mar 5;9(3):e89982. doi: 10.1371/journal.pone.0089982 (PMC3943853; doi:10.1371/journal.pone.0089982)
Supplement: Figure S2 — Confidence intervals of coefficients of regression models of emigration patterns. (PDF) [file pone.0089982.s009.pdf]

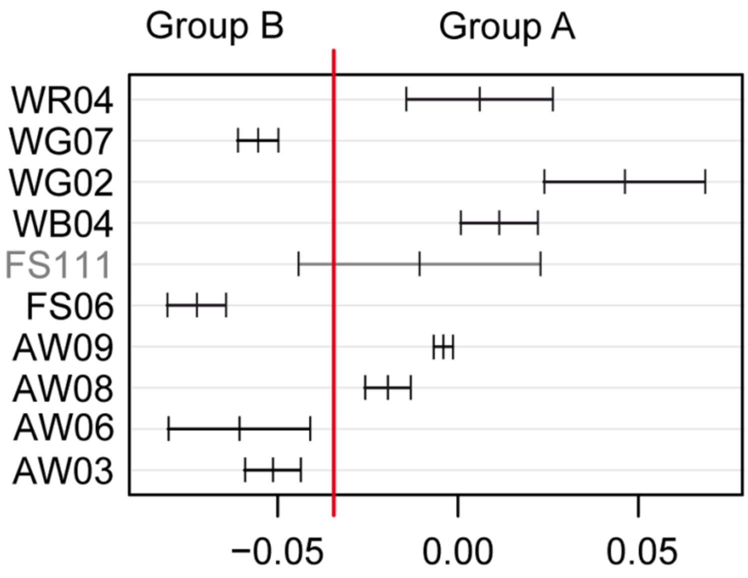

Figure S2. Confidence intervals on coefficients of regression models describing metamorphic weight in relation to development time (see red line Fig. 4, Table S3). Pond FS111 was excluded from further analysis to accentuate the difference between slope values in the two groups.
